# Major Stressors Favoring Cholera Trigger and Dissemination in Guinea-Bissau (West Africa)

**DOI:** 10.3390/ijerph182111296

**Published:** 2021-10-27

**Authors:** Ana Machado, Eva Amorim, Adriano A. Bordalo

**Affiliations:** 1 Laboratory of Hydrobiology and Ecology, Institute of Biomedical Sciences Abel Salazar (ICBAS—UP), University of Porto, Rua Jorge Viterbo Ferreira 228, 4050-313 Porto, Portugal; ecamorim@icbas.up.pt (E.A.); bordalo@icbas.up.pt (A.A.B.); 2 Interdisciplinary Centre of Marine and Environmental Research (CIIMAR—UP), University of Porto, Novo Edifício do Terminal de Cruzeiros do Porto de Leixões, Avenida General Norton de Matos, s/n, 4450-208 Matosinhos, Portugal

**Keywords:** cholera, waterborne diseases, WASH, Guinea-Bissau, Sub-Saharan Africa

## Abstract

Cholera remains a heavy burden worldwide, especially in Sub-Saharan African countries, which account for the majority of the reported cases on the continent. In this study, a 27-year retrospective analysis of cholera epidemics in Guinea-Bissau was performed in order to highlight major stressors fueling the trigger and dissemination of the disease. Although the role of environmental factors did not always have the same degree of importance for the onset of epidemics, a cholera seasonal pattern was clearly perceived, with most of the reported cases occurring during the wet season. The generated theoretical hypothesis indicated rainfall above climatological average, associated with a lack of WASH (water, sanitation and hygiene) infrastructure, and the occurrence of concomitant epidemics in neighboring countries as the key indicators for optimal conditions for cholera to thrive in Guinea-Bissau. Warmer air temperature, the increase in sea surface temperature, and the decrease in salinity in the coastal areas may also contribute to the emergence and/or aggravation of cholera events. Prediction of the conditions favorable for cholera growth and identification of risk pathways will allow the timely allocation of resources, and support the development of alert tools and mitigation strategies.

## 1. Introduction

Since the turn of the century, there has been an increasing concern with identifying and addressing the factors driving water-associated infectious diseases observed worldwide [1]. With millions of cases emerging every year, cholera, an acute, secretory diarrhea disease transmitted by the consumption of contaminated drinking water and food, still poses a serious health problem worldwide, especially in low- and middle-income countries. Africa, alone, accounts for the large majority of the worldwide officially notified cholera cases and associated deaths each year, with a cholera fatality rate (CFR) of 2.28%, which is nearly double the global average (~1%) in non-African regions [2]. Sub-Saharan African countries reported 83% of the cholera cases between 2000 and 2015 [3]. The situation could be even more pressing since the WHO estimates that the officially reported cases correspond to only 5–10% of the annual effective cases [4]. In order to tackle the problem, in 2017, the Global Task Force on Cholera Control (GTFCC) launched a global strategy on cholera control, Ending Cholera: A Global Roadmap to 2030, with the objective of reducing cholera deaths by 90% worldwide [5]. Moreover, this multisectoral approach is closely associated with the United Nations Sustainable Development Goals, to ensure access to clean water and sanitation for all, to reduce existing inequities, and to promote healthy lives and well-being at all ages [6].

*Vibrio cholerae*, the etiological agent of cholera, is an autochthonous bacterium in marine, coastal, and freshwater environments. Therefore, human exposure to potential pathogenic *Vibrio* species can be mitigated but not eliminated. Although high-population areas with poor access to safe drinking water and without sanitation contribute to the emergence and spread of cholera, unequivocal evidence that environmental factors have a concomitant major influence on the disease dynamics is now established [7,8,9,10]. This influence on disease dynamics can either be direct or realized through the control of other organisms that serve as reservoirs for the bacterium, such as copepods [9,11,12]. Several studies have demonstrated the association between environmental parameters and the survival and dynamics of *V. cholerae* in the environment [13,14,15,16,17], with special attention given to temperature and salinity.

In addition to the promotion of access to potable water and sanitation, another powerful action to decrease cholera incidence seems to be the establishment of a robust surveillance approach [2,18]. Due to the link between environmental factors and cholera, in the last decades, the development of mathematical models to predict cholera emergence in endemic regions based on environmental monitoring have earned special relevance. The design of such alert tools, optimized to regional conditions, is essential to perform risk analysis, to make decisions and manage resources (especially in impoverished countries), and to reduce the impact of potential cholera epidemics. Owing to these observations, eventual linkages between environmental signatures and cholera epidemics began to be studied, resorting to theoretical models for specific areas in Asia, South America, and Africa [19,20,21]. In the particular case of Africa, studies explored and found associations between the number of cholera cases and air temperature, sea surface temperature, and rainfall in Southeastern Africa area, as well as for single countries, such as Ghana, Zambia, Senegal and Zimbabwe [7,20,22,23,24].

In spite of the evidence for the need to examine the impact of climatic factors on cholera in Africa [22,25], the knowledge is still scant, particularly regarding Sub-Saharan Africa and at regional levels. This lack of knowledge is even more striking since Africa takes on the burden of the heavier impact, with the majority of cholera cases reported worldwide [3]. Indeed, since the seventh cholera pandemic reached Africa in 1970, the disease has become endemic in many African countries, remaining a recurring cause of large, deadly, multinational epidemics in West, Central, and East Africa [26].

In this vein, the objective of the present study was to assess major stressors responsible for the trigger and dissemination of the disease in the Sub-Saharan West African country of Guinea-Bissau, one of the poorest countries in the world. Special attention was given to the association between environmental variables and cholera cases in order to evaluate the potential to predict cholera epidemics and to construct alert tools based on environmental monitoring.

## 2. Materials and Methods

### 2.1. Study Area

West-coast African Guinea-Bissau is one of the poorest countries in the world, according to the Human Development Index (rank 178 out of 189 countries in 2019). Currently, with 1,816,000 inhabitants (about 27% in the capital, Bissau), the life expectancy at birth in Guinea-Bissau in 2016 was 58 and 61 years for males and females, respectively. Moreover, 82 children die under the age of five per 1000 live births (2018) [3]. Officially “improved” water sources (not necessarily meaning potable water) are accessible to 74% of the population, and only 18% of the population has access to proper sanitation [27]. The majority of the people use open/unprotected, shallow, hand-dug wells (<15 m) as the sole source of water for daily needs, including drinking water. From those sources, 80% are contaminated with fecal material and have an acidic pH [28]. The population defecates in latrines whose distance from wells is often insufficient to avoid contamination of the well water with human pathogenic microorganisms. Additionally, domestic animals, such cows, goats, chickens, and pigs, wander freely in the vicinity of the wells [29]. The population is very vulnerable to disease (malaria, diarrhea, respiratory diseases, HIV, and malnutrition), and the health system is fragile and not universally available.

### 2.2. Epidemiological Data

The incidence of cholera analyzed in this study, related to the annual number of cholera cases reported in Guinea-Bissau and the cumulative incidence by sector level for the 1986–2013 period, was compiled from the *WHO Global Health Atlas* [3] and the *Weekly Epidemiological Record* [4,30,31,32,33,34,35,36,37,38], from the *Plan strategique de prevention et de riposte contre le cholera en Guinee Bissau* 2009–2013 [39], and *The time series analysis of cholera in Guinea-Bissau*, 1996–2008 report [40].

### 2.3. Environmental Data

Sea surface temperature (SST) and salinity data for the period 1986 to 2013 were acquired from the monthly mean global ocean physics reanalysis (CGLORS) dataset, available on the Copernicus Marine Environment Monitoring Service (CMEMS) website (http://marine.copernicus.eu/, accessed on 2 June 2020). Data were extracted from selected pixels (10.9–12.16° N, 17.25–15.5° W) with a 0.25 degree resolution in order to represent the sea surface along the Guinea-Bissau coast.

Air temperature and rainfall data for the 1986–2013 period were extracted from the historical GHCN gridded V2 dataset provided by NOAA/OAR/ESRL PSD, available on their website (http://www.esrl.noaa.gov/psd/, accessed on 4 June 2020). The resolution was 0.5 degrees for air temperature and 2.5 degrees for rainfall for the available grid of pixels covering the Guinea-Bissau country area (11–13.5° N, 18–14° W).

Sea surface and air temperature are reported in degrees Celsius; salinity in the dimensionless practical salinity scale, defined as conductivity ratio; and rainfall in mm. The annual average dataset of each environmental parameter is shown in Table S1.

### 2.4. Statistical Analysis

The descriptive analysis of the cholera cases time series and satellite-derived environmental variables under scrutiny was performed using scatter plots.

For environmental factors, percent deviation of monthly anomalies was determined following Jutla et al. [23]. Hence, monthly anomalies were calculated by subtracting the month value from the average of the 27 years of monthly data. The percentage deviation resulted from the ratio between the monthly anomaly and the 27-year average value for the respective factor. A positive anomaly inferred a value higher than the average for 27 years.

The Spearman’s rank correlation coefficient was used to assess the relationship between environmental factors and the number of cholera cases in Guinea-Bissau. Concerning the correlation coefficient, the subscript abbreviation letters stand for the specific environmental parameter (R—rainfall, SST—sea surface temperature, Sal—salinity), and the numbers for the month lag applied (0—no Lag, 1—one month, 2—two months). The significance level used for all tests was 0.05. The statistical analyses were performed using R v3.5.0 [41].

The crude, non-adjusted odds ratio and the respective 95% confidence interval (CI) were calculated, according to Altman 1991 [42], as the ratio between the probability that a cholera event will occur following exposure to the trigger factor and the probability that an event will not occur. The odds ratio (OR) concerning the strength of association between the WASH factors and cholera transmission were retrieved from the literature [43] and reported for the Guinea-Bissau conditions.

## 3. Results and Discussion

### 3.1. Cholera in Guinea-Bissau Time Series Annual Overview

**Figure 1:**
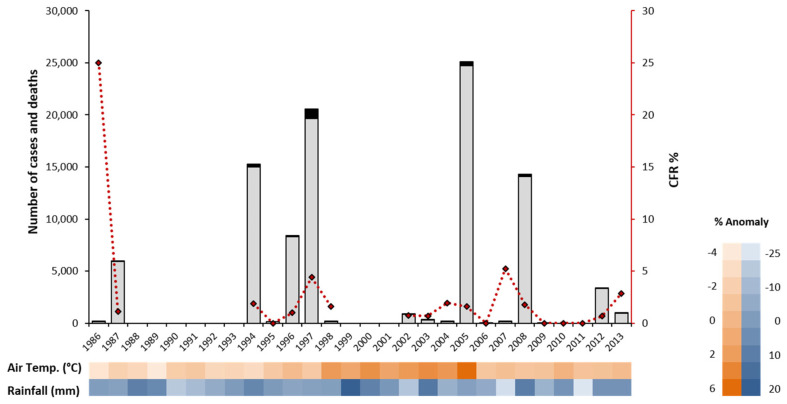
Number of cholera cases, deaths, and case fatality rate (CFR) in Guinea-Bissau between 1986 and 2013, from the WHO weekly epidemiological reports. Bar height corresponds to the total number of cholera cases reported; black portion of the bars corresponds to number of deaths. The red dashed line corresponds to the CFR percentage. Heat map (ascending color gradient) showing air temperature (orange, annual average range 27.69–30.57 °C) and rainfall (blue, annual average range, 844–1345 mm) deviation to the 27-year time series average.

Cholera is endemic in Guinea-Bissau [44], being first officially stated in 1986, although the Portuguese army colonial medical services, in 1973, had already referred several cases in the hinterland. Ever since, major epidemics have periodically occurred, in 1987, 1994, 1996, 1997, 2005, 2008, and 2012 (Figure 1). A clear increase in cholera outbreaks was noticed from the mid-1990s onwards. Not only was a higher number of epidemic events reported, but a higher attack rate within each epidemic was also registered. After 2008, a decrease in the number of cases reported was noted, with almost no cholera cases for three consecutive years (Figure 1). Within the 1986–2013 time frame, Guinea-Bissau reported 95,946 cholera cases, with 2117 associated deaths and a case fatality rate of 2.2%.

The missing data in the time series presented were due to the absence of reported cases during the periods of political instability and civil war (1988–1993 and 1998–2001), with consequent disruption of the already fragile health and surveillance systems existing in the country. Evidently, these gaps are a limiting factor in the analysis of such time series.

Through simple visual inspection of the annual time series, it was possible to observe an association between cholera incidence and key environmental variables, namely air temperature and rainfall (Figure 1). At this level, the identified general trend was an increase in the number of cholera cases, leading to an epidemic event, when an increased air temperature and/or rainfall for the same year was present. Therefore, it could be observed that rainfall was above the 27-year time series average during the 1994, 2008, and 2012 epidemics (8, 12 and 4%, respectively), whereas in the 2005 epidemic, the annual average air temperature was 6% higher than the average.

### 3.2. Spatial Distribution

**Figure 2:**
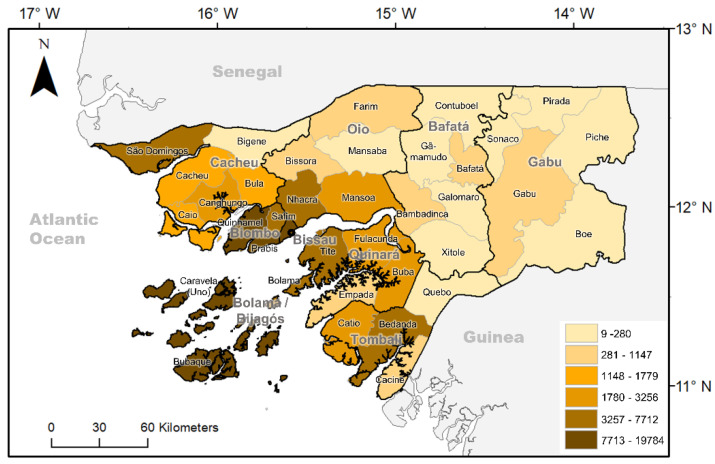
Geographical distribution of cholera cumulative incidence per 10,000 inhabitants by sector level in Guinea Bissau, 1986−2013. Source: Cholera Platform. (http://www.plateformecholera.info/attachments/article/231/GNB_incidence%20map_PhatChol.pdf, accessed on 7 September 2020).

The majority of the cholera cases declared in Guinea-Bissau from 1986 to 2013 have been reported in three main areas: Bissau (capital), Biombo (Quinhámel, Prabis, Safim), close to the capital, and the Bijagos Islands (Figure 2). Cross-border outbreaks frequently occurred between Guinea-Bissau and the neighboring countries, Senegal and Guinea. Indeed, all the major epidemics in Guinea-Bissau were accompanied by epidemic events in Senegal and/or Guinea (Table 1).

The coastal zone, very permeable to transboundary movement of people, and urban, densely populated areas, such the capital, Bissau, appear to be hotspots, with higher risk for potential cholera outbreaks. National and Senegalese or Guinean migrant fishermen, seasonal farmers, and tradespeople have been referenced as increased-risk populations [45]. Similarly, traditional animist funeral rituals and consumption of water sachets are reported as practices that may facilitate the spread of cholera [45,46].

### 3.3. Historical Cholera Epidemics in Guinea-Bissau

In this section, we will describe the dynamics of the historical epidemics in Guinea-Bissau that account for more than 10,000 cases, as well as the most recent one (Table 1). In the 1990s, in the aftermath of years of political instability, three large-scale epidemics struck the country in 1994, 1996, and 1997.

The first epidemic of the decade started in October 1994, in the Bijagos Archipelago, and spread rapidly to the rest of the country, to end by January 1995, 14 weeks later. During this epidemic 15,878 cases and 306 deaths were reported [47]. The country’s overall CFR was 1.8%. The epidemic had its peak in November (at the onset of the dry season), and the region of Biombo had the highest recorded cholera incidence outside Bissau, with a CFR of 3.7%. *V. cholerae* O1, serotype Ogawa, biotype E1 Tor, was isolated from patients suffering from cholera [48]. The dissemination of cholera seemed to be associated with funeral rituals [48]. Furthermore, the epidemic followed a major epidemic in the neighboring southern country Guinea (31,415 cases) and in the war-torn Sierra Leone (46,000 cases). Moreover, molecular studies confirmed the epidemiological results of cholera case migration between Sierra Leone and Guinea [49].

From 1996 to 1998, two consecutive epidemics were recorded in Guinea-Bissau, accounting for 26,959 cases and 961 deaths. The first epidemic began in October 1996 in the capital, Bissau, lasting 30 weeks, throughout the dry season, ending in April 1997 (Figure S1). The number of cholera cases rose to 10,844, with 108 reported deaths, and the country estimated attack rate (AR) and CFR were 1%. Two peaks could be observed during the epidemic: in week 46 of 1996 and in week 1 of 1997, with 798 and 658 cases, respectively. Although cases were reported nationwide, with the exception of the Bijagos Islands, the most affected regions were, once again, Bissau (AR 4%) and Biombo (AR 2.5%).

The second epidemic was a resurgence of the previous one, starting in May 1997 at the onset of the wet season, and ended in January 1998 (38 weeks). The number of notified cholera cases in the country was 16,115, with 853 deaths and an overall estimated AR of 1.4% and a CFR of 5.3%. The epidemic peak could be observed in week 32 (in the rainiest month of the year), with 995 cases, and a smaller second peak could be perceived from epidemic weeks 37 to 40 (Figure S2). The outbreak started in the capital, Bissau (AR 8.2%), and spread countrywide during the second semester of 1997, affecting mainly the nearby Biombo region (AR 4.2%) and the islands of the Bijagos Archipelago (AR 4.6%).

After a lull period with and absence of or low cholera cases reported, in June 2005, a major epidemic struck the country, accounting for 25,282 cases and 397 deaths. This epidemic had a duration of 35 weeks, ending by February 2006, with an estimated AR and CFR of 1.8% and 1.6%, respectively. The epidemic curve showed a peak in week 34, late August of 2005, coinciding with the peak of the wet season, with 2106 cases and a second peak in weeks 40 and 41, accounting for 1200 cholera cases (Figure S3). Similarly to previous epidemics, the outbreak started in Bissau and spread nationwide, with the coastal zones once again highly affected, particularly Biombo (AR 6.1%) and the Bijagos Islands (AR 9.6%). This epidemic was part of major outbreaks occurring in Africa during that period and followed the trend of the northern neighboring country of Senegal, with 31,719 cholera cases. The 2005 Senegal cholera epidemic was the largest recorded epidemic by the WHO for Africa, the dissemination of which was a result of a traditional pilgrimage that draws one to two million people to the holy city of Touba [50].

The latest large epidemic ravaged the country between May 2008 and January 2009 (35 weeks). A total of 14,228 cases and 225 deaths were reported. The estimated AR was 0.9%, and the CFR was 1.6%. The epidemic curve peak was observed in week 40, with 1391 cases (Figure S4). The first cases were reported in the southern region of Tombali, close to Guinea, with subsequent spread nationwide. The most affected areas were the coastal regions of Bissau (AR 2.3%), Biombo, and the Bijagos Islands (AR 3.0%).

Between 2012 and 2013 (67 weeks), a smaller epidemic in Guinea-Bissau, accounting 4249 cases and 50 deaths, was reported. This outbreak started in the heavily populated Missira neighborhood in Bissau [45]), in late August, and spread throughout the country. However, all recorded cases in 2013 were in the Tombali coastal region, on the border with Guinea. The estimated overall AR was 0.3%, and the CFR was 1.2%, with the epidemic peak reported in week 46 (at the onset of the dry season), with 442 cases (Figure S5). Once again, the coastal zones were the most affected areas, with the Bijagos Islands showing the highest attack rate (AR 7.68%), and Oio region with the highest CFR (10.7%). Furthermore, this epidemic was linked to the ones occurring in Sierra Leone and Guinea during the same time period.

### 3.4. Relationship between Environmental Factors and Cholera Epidemics

**Figure 3:**
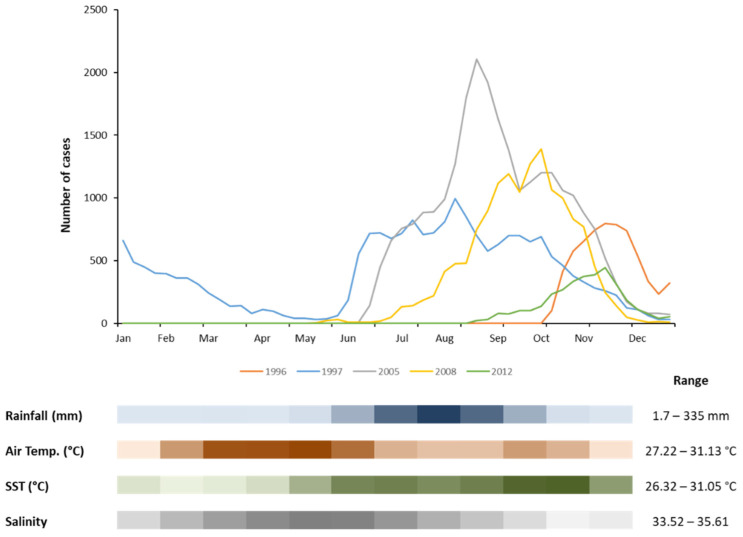
Weekly number of cholera cases during the major epidemics occurring in Guinea-Bissau between 1986 and 2013. Heat map (ascending color gradient) showing average monthly rainfall (blue), air temperature (brown), sea surface temperature (SST, green), and salinity (grey) over the 27-year time series.

The seasonal pattern of the major cholera epidemics revealed that most of the epidemics occurred during the wet season (June to November), displaying a steep decrease in the number of reported cholera cases from December onwards (Figure 3). Two onset periods have been identified. The first started at the beginning of the wet season, between May and July, leading to the heaviest epidemics in the country. The second occurred in the second half of the wet season, in September. Furthermore, within the country, different regions showed slightly different seasonal patterns, with an increased risk from the beginning of April in Biombo, mid-April in Bissau, and May in the Bijagos Islands (Figure 2).

In order to further understand the factors triggering cholera epidemics in the country, the relationship between climatic factors essential to the survival of the cholera etiological agent (*V. cholerae*) and monthly reported cholera cases has been investigated (Figure 3).

From the analysis of the 27-year monthly time series average of key environmental constraints, it was possible to recognize that most cholera outbreaks leading to severe epidemics traditionally started with the first rains. An increment in the number of cases could be observed throughout the wet season, peaking in August–September, with the heaviest rains. Indeed, rainfall can have an impact on the cholera outbreak onset, since is responsible for the increment of water turbidity, a consequence of the flow of soil particles into the coastal zone. The relationship between turbidity and bacterial abundance, including *Vibrio* spp., has been previously reported, with sediments and suspended particulate matter as sources and reservoirs of nutrients, as well as an attachment surface for vibrios [51,52]. Rainwater infiltration and percolation are also accountable for the transport of suspended particles with associated bacteria from the nearby environment (latrines, waste dumps) to the well water used for drinking by the majority of the population in Guinea-Bissau [28,29]. In this way, the contamination of the groundwater is of particular importance not only for the epidemic onset but also in its escalation and propagation. *V. cholerae* excreted in the feces of patients reach untreated well water, closing the cycle and potentially infecting an exponential number of persons. In fact, *V. cholerae* have been found in well water used for drinking in Guinea-Bissau even during non-epidemic periods [53].

The outbreak onset for the major epidemics seems to occur in warmer months, with air temperatures above 30 °C (Figure 3), even though the lower monthly average air temperature observed in Guinea-Bissau was above 27 °C. The association between water temperature and salinity and the abundance of *Vibrio* spp., including *V. cholerae*, in the environment is well documented [13,54]. Being a coastal country, the outbreaks in Guinea-Bissau seem to develop when the sea surface temperature increases and salinity starts to decline (Figure 3). Indeed, temperature plays a pivotal role in bacterial abundance and distribution, and values above 20 °C can stimulate growth [52]. Furthermore, vibrios can enter a viable but nonculturable state (VBNC) when the environmental conditions are not favorable, and temperature is known to be important in reversion from that VBNC state [55]. However, in tropical environments, where the optimal temperature for growth is roughly maintained throughout the year, salinity emerges as an important controller for *Vibrio* dynamics regulation [56,57]. In these conditions, even a modest decrease in salinity has been shown to promote *Vibrio* abundance [13,16,58].

The empirical observations are corroborated by the statistically significant correlations (*p* < 0.05, Figure S6) found between the monthly number of cholera cases and the climatic factors studied. Positive correlations were observed between cholera cases and a time lag of one to two months in rainfall (r_R1_ = 0.22 and r_R2_ = 0.29, respectively) and up to two months in sea surface temperature (r_SST_ = 0.27, r_SST1_ = 0.32 and r_SST2_ = 0.28). Moreover, a significant negative correlation of up to one-month time lag was detected with coastal water salinity (r_Sal_ = −0.29, r_Sal1_ = −0.18). It should be noted that, as expected, salinity and rainfall were inversely correlated (r_Sal_—_R1_ = −0.33, r_Sal_—_R2_ = −0.71). Indeed, this dynamic has been already described for *Vibrio* spp. in Guinea-Bissau coastal waters [16], with higher *V. cholerae* abundance observed when salinity was lower, and SST was higher.

**Figure 4:**
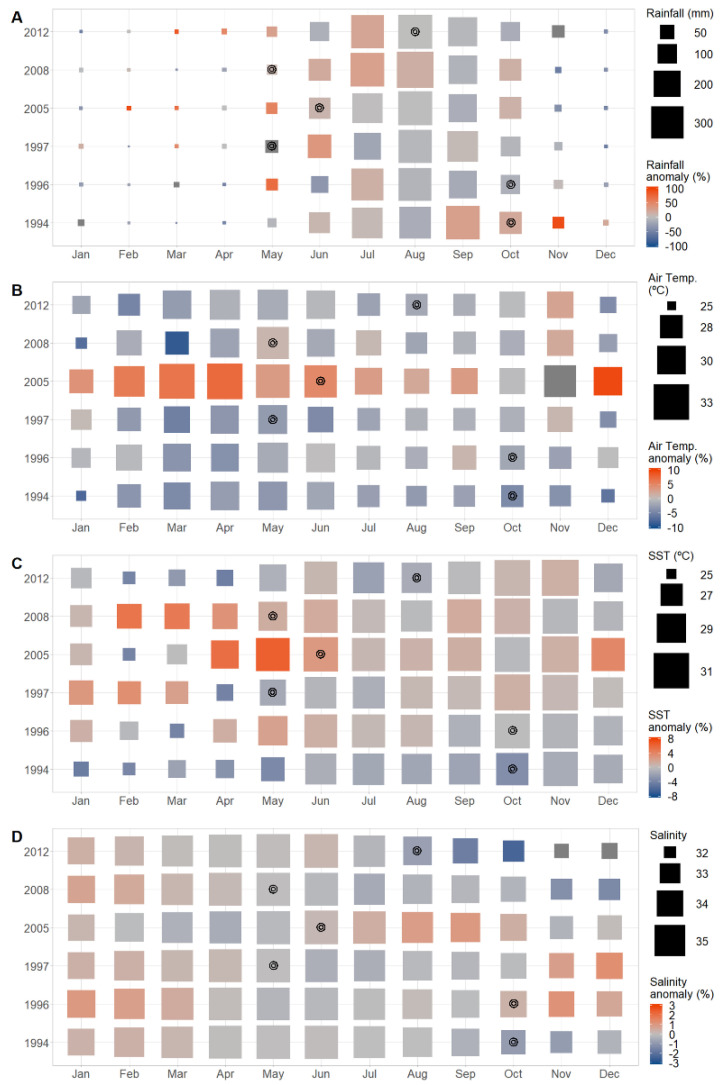
Temporal variation of the environmental parameters for the major cholera epidemic years between 1986 and 2013 in Guinea-Bissau. (**A**) rainfall; (**B**) air temperature; (**C**) SST; (**D**) salinity. Heat map shows the deviation of each parameter from the 27-year average. Circles indicate the month of cholera onset for each epidemic.

Despite the evident contribution of environmental parameters to the development of the cholera epidemics in Guinea-Bissau, no secular trend could be found. In this way, the epidemics’ onsets appear to be conditioned by several environmental factors that can have different degrees of importance, according to the time period (Figure 4).

The 1994 epidemic (moderate El Niño year) started in October, after 4 months of water temperatures above 29 °C and when salinity decreased below 34, and conditions were favorable for the survival of *V. cholerae* in coastal waters. The epidemic started by the end of the wet season, following 5 months of heavy downpour, with rainfall above the climatological average for the start month and the previous one (20 and 28%, respectively). The same conditions were verified in the following 1996 epidemic (a moderate La Niña year), with the exception that the amount of rain observed was not above the climatological average, but the air temperature was slightly (1%) above average in the month preceding the onset. The 1997 epidemic (a very strong El Niño year) started in the aftermath of the previous one, simultaneous to the onset of the wet season, whose rainfall amount in May was above the climatological average by 106%. The major epidemic of 2005, a weak El Niño and La Niña year, emerged in June during the third consecutive month of climatological water temperatures above average (6, 6, and 3%, respectively) and salinity marginally below average. Additionally, the recorded air temperature was above climatological average (up to 7%) since the beginning of the year, and rainfall was 54% above average in the month previous to the onset, and about 9% for June. The 2008 epidemic (a strong La Niña year) had its beginning in May, the hottest month of that year, with air temperatures averaging 31.4 °C (about 1% above the climatological average) and water temperatures also above average since the beginning of the year (up to 5%). Again, the onset coincided with the beginning of the wet season, with rainfall above average by 9% for that month.

In the most recent 2012 epidemic (moderate La Niña year), the first cases were reported in August 2012, the fourth month within the wet season and the rainiest month recorded for the year. Rainfall amounts above the climatological average by about 23% were registered in the previous month of July.

### 3.5. Theoretical Model of the Influence of Different Stressors on Cholera Trigger and Transmission

**Figure 5:**
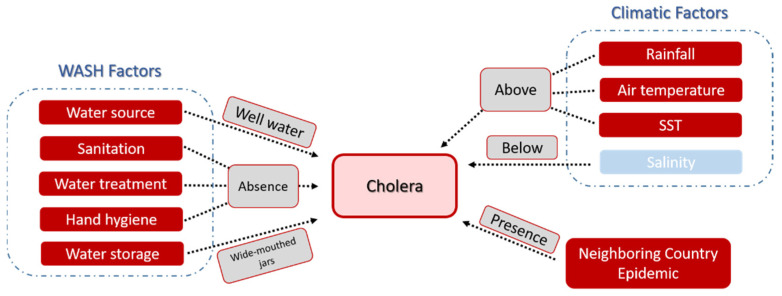
Diagram showing the adaptation to Guinea-Bissau of the influence of different stressors on cholera transmission. Red and blue boxes indicate positive and negative relationships, respectively. SST—sea surface temperature.

We present a theoretical model (Figure 5) displaying the influence of different stressors on cholera trigger and transmission for Guinea-Bissau, including both hydroclimatological and societal factors. Different outcomes are possible if different factors are fed, with synergetic effects occurring between them, as in the case of the 2005 epidemic, where all the factors converged, generating a major epidemic. Similarly to the reports from other African countries [7,20,23], in Guinea-Bissau, rainfall seems to be a major driving factor in the development of a cholera epidemic, with the onset and/or dissemination occurring seasonally, concomitantly with the wet season. Rainfall above the climatological average for the onset or the previous month showed higher odds (OR 4.6; 95% IC 0.22–97.5) of resulting in a major cholera epidemic. Although air temperature, SST, and salinity showed a correlation with the incidence of cholera cases, proving to have a role in the development of particular cholera epidemics, no increment in the odds occurrence of major epidemics could be observed.

It should be stressed that the occurrence of cholera outbreaks in neighboring countries increases the probability of a cholera epidemic in Guinea-Bissau. The odds of reported cholera cases in Guinea-Bissau are greater when cholera cases have also been reported in the neighboring countries (OR 1.8; 95% IC 1.07–3.14). Indeed, all major epidemics were concurrent to events in Senegal and Guinea, showing the importance of cross-border movement of people between neighboring countries for the dissemination of cholera (human to the environment). The importance of effective transboundary surveillance for the control of cholera epidemics has been already recognized in other studies concerning epidemiological and molecular surveys in neighboring countries [49]. The inevitable interaction between a given population and daily use of contaminated water increases the probability of development of disease, and consequently, the occurrence of epidemics. However, in the absence of this interaction and even in the presence of other stressors keen for cholera transmission, the occurrence of cholera declines, as already reported [23].

Since no epidemiological studies were available for Guinea-Bissau concerning the causal effect between WASH factors and the transmission of cholera, the associated relationship was retrieved from the literature regarding low- and middle-income countries [43] and reported for the local conditions. The majority of the population retrieves water for daily use, including drinking, from shallow wells that are easily prone to contamination, especially during periods of heavy rainfall [28]. The use of well water as a water source for domestic use was identified as a factor increasing the odds (OR 3.01; 95% CI 1.08–8.39) of cholera transmission. Additionally, the use of untreated water (no chlorination, boiling, or solar disinfection) was also found to result in higher odds for cholera transmission (OR 2.8; 95% CI 1.82–4.29). In Guinea-Bissau, only 18% of the population has access to sanitation [27], a fact that can be associated with the contamination of their shallow water sources and the consequential development of cholera. In homes, water is traditionally stored in 40–45 L earthen wide-mouthed jars, with the intent to keep it fresh. A communitarian 0.5 L stainless steel or plastic mug is inserted by hand into the container to retrieve the water for consumption. The use of no narrow-mouthed containers to store water was shown to relate with higher odds of cholera transmission (OR 2.02; 95% CI 1.15–3.57). Defecating in the open and shared sanitation facilities (latrines) has also been reported to contribute to higher odds of cholera transmission (OR 2.64; 95% CI 1.58–4.39 and 1.82; 95% CI 1.33–2.51, respectively). Hand hygiene (before food handling, after defecation) can contribute to lower odds of cholera transmission (OR 0.45; 95% CI 0.32–0.65 and OR 0.28; 95% CI 0.17–0.45, respectively). Although there are no published studies available on this subject for Guinea-Bissau, it can be hypothesized, as a result of the perception of the authors in the field, that the scarcity of potable water and the high level of contamination of water sources in the country leads to the absence or inefficiency of hand-hygiene procedures in daily routines.

Although rather conservative, this study and the generated theoretical model possess inherent limitations associated with the data quality, essentially owing the lack of a robust surveillance system and overall health system in Guinea-Bissau. Moreover, due to the complexity of ecological systems, the inherent collinearity often found among explanatory variables frequently poses serious problems for statistical and inferential analyses, which can lead to misinterpretation of results. In our study, the proposed explanatory variables are not only correlated with each other but also temporarily autocorrelated, and therefore, it is not possible to separate the effects or contributions of each individual variable to the number of cholera cases from one another. Furthermore, the study was based on country-level epidemiological and environmental data. However, considering the already-known cholera hot spots, highlighted earlier, country asymmetries in the degree of risk are expected. In this vein, further studies are needed to attune the theoretical model to regional and community levels.

Despite its limitations, this study sheds light on the hitherto unknown environmental and societal factors implicated in the trigger and dissemination of cholera epidemics in Guinea-Bissau, that periodically ravage the Sub-Saharan coastal country and clearly defines pathways to diminish the risk incidence. The improvement of existing, albeit fragile, WASH infrastructure is the most efficient way to tackle the cholera problem, with particular emphasis on prevention. Since environmental factors are impossible to eradicate or control, effective monitoring of these factors is the best way to predict cholera events. Moreover, the importance of cross-border movement stands out as a major factor that can easily be solved with national surveillance implementation. International cooperation on surveillance, between neighboring countries, could be a powerful tool to implement as a strategy in the prevention and mitigation of cholera epidemics. In this way, the adoption of simple steps, in line with the lack of funds, infrastructure, and human resources, can allow for the timely allocation of assets, decreasing the cholera burden in low- and middle-income countries.

## 4. Conclusions

The major stressors responsible for the trigger and dissemination of cholera were identified based on historical analysis of cholera epidemics in Guinea-Bissau. A theoretical model was developed to integrate information on hydroclimatological and societal factors to estimate the risk of cholera in the country. Heavy rainfall above the climatological average seems to be the main factor controlling cholera dynamics since it enhances the exposure to contaminated water sources. Additionally, increased air and sea surface temperatures, and low salinity contribute to *V. cholerae* survival and dissemination in costal ecosystems, favoring the occurrence of cholera events. The lack of proper WASH infrastructure in the country was recognized as key enabling factor for the development of cholera epidemics. If societal factors are preserved, and even if environmental conditions are favorable, the risk of occurrence of a cholera epidemic is reduced. Furthermore, since all major cholera epidemics in Guinea-Bissau were concomitant with epidemics in neighboring countries, the results strongly suggest a robust cross-border surveillance in order to avert and restrain the emergence of disease. In spite of inherent limitations, the construction of integrative risk models can allow for forecasts that may help to plan and manage public health decisions, and ultimately save lives. Timely allocation of resources, such as health professionals and equipment, is pivotal to prevent or limit the widespread dissemination of future epidemics, especially in impoverished and vulnerable countries such as Guinea-Bissau.

## Figures and Tables

**Table 1:** Epidemiological parameters of cholera outbreaks in Guinea-Bissau between 1986 and 2013.

Year	Cases	Deaths	1st Case		Epidemic Extension	Cases	
			Date	Region		Senegal	Guinea
1986	200	50	October	-	Northern province *	426	286
1987	6000	68	September	Bissau	Coastal regions	2757	-
1994	15,878	306	October	Bijagos	Country level	-	31,415
1996/1997	10,844	108	October	Bissau	Country level except Bijagos	16,107	287
1997/1998	16,115	853	May	-	Country level	371	-
2002/2003	1132	8	December	Bissau	Bissau	-	67
2004	227	3	October	Bijagos	Bijagos	1227	1516
2005	25,282	397	June	Bissau	Country level	31,719	3821
2007	73	3	October	Tombali	Tombali	3984	8546
2008	14,228	225	May	Tombali	Country level	1283	513
2012	3280	22	August	Bissau	Country level	-	7325
2013 ^+^	969	28	January	Tombali	Tombali	-	319

* Northern province—Biombo, Cacheu, Oio regions. + The 2012 epidemic was not declared over, so the 2013 epidemic is a prolongation of the previous 2012 one.

## Data Availability

All data generated or analyzed during this study are included in this published article [and its supplementary information files]. Notwithstanding that, the datasets used and/or analyzed during the current study are available from the corresponding author on reasonable request.

## Supplementary Materials

The following are available online at https://www.mdpi.com/article/10.3390/ijerph182111296/s1. Table S1: Annual average of the environmental parameters for the studied time series period; Figure S1: Weekly number of cholera cases in Guinea-Bissau between January 1996 and April 1997; Figure S2: Weekly number of cholera cases in Guinea-Bissau between May 1997 and January 1998; Figure S3: Weekly number of cholera cases in Guinea-Bissau between June 2005 and February 2006; Figure S4: Weekly number of cholera cases in Guinea-Bissau between May 2008 and January 2009; Figure S5: Weekly number of cholera cases in Guinea-Bissau between August 2012 and November 2013; Figure S6: Matrix of Spearman correlations between the monthly number of cholera cases and the environmental variables.
