# Supplementary material for: Major Stressors Favoring Cholera Trigger and Dissemination in Guinea-Bissau (West Africa)
Source: Int J Environ Res Public Health. 2021 Oct 27;18(21):11296. doi: 10.3390/ijerph182111296 (PMC8583644; doi:10.3390/ijerph182111296)
Supplement: Supplementary file 1 [file ijerph-18-11296-s001.zip › ijerph-1352089-supplementary.pdf]

Major Stressors Favoring Cholera Trigger and Dissemination in Guinea-Bissau (West Africa)

Ana Machado[1, 2]*, Eva Amorim[1, 2], Adriano A. Bordalo[1, 2]

[1]ICBAS—UP, Institute of Biomedical Sciences Abel Salazar—University of Porto, Rua Jorge Viterbo Ferreira 228, 4050-313 Porto, Portugal

[2]CIIMAR—UP, Interdisciplinary Centre of Marine and Environmental Research of the University of Porto, Novo Edifício do Terminal de Cruzeiros do Porto de Leixões, Avenida General Norton de Matos, S/N, 4450-208 Matosinhos, Portugal

**ammachado@icbas.up.pt*

Table S1. Annual average of the environmental parameters for the studied time series period. SST - sea surface temperature.

Year	Air temperature (ºC)	Rainfall (mm)	SST (ºC)	Salinity
1986	27.79	1120	27.87	35.04
1987	28.25	1106	28.82	34.87
1988	28.11	1229	28.26	34.96
1989	27.69	1191	28.49	34.84
1990	28.35	955	28.34	34.87
1991	28.46	1004	28.11	34.68
1992	28.12	1066	28.27	34.69
1993	28.18	1127	28.55	34.58
1994	28.01	1204	28.17	34.61
1995	28.45	1131	28.63	34.80
1996	28.74	1094	28.84	34.76
1997	28.44	1106	28.93	34.70
1998	29.50	1113	28.88	34.86
1999	29.20	1345	28.16	34.69
2000	29.72	1223	28.75	34.39
2001	29.26	1143	29.09	34.47
2002	29.51	969	28.73	34.61
2003	29.79	1258	28.52	34.74
2004	29.57	1049	28.79	34.89
2005	30.57	1111	29.29	34.71
2006	28.51	1070	28.95	34.52
2007	28.67	876	28.79	34.79
2008	28.52	1238	29.24	34.58
2009	28.57	1038	28.34	34.57
2010	28.93	1156	29.46	34.23
2011	28.59	844	28.96	34.52
2012	28.63	1160	28.51	34.32
2013	28.74	1160	30.02	34.29
**27-years average**	**28.75**	**1110**	**28.71**	**34.66**

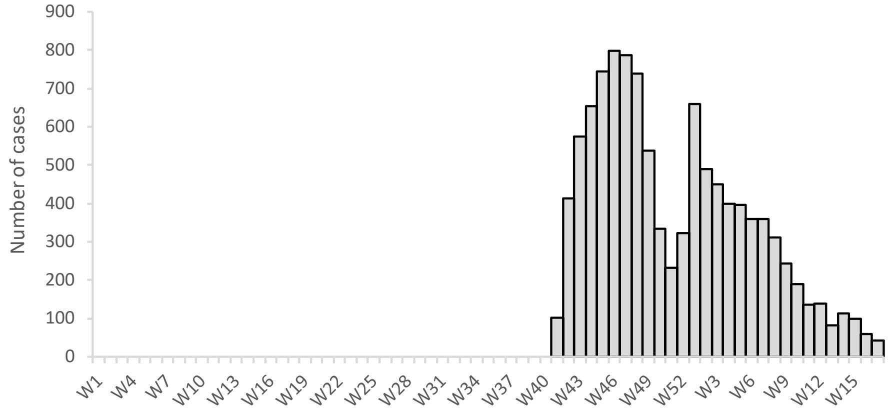

Figure S1. Weekly number of cholera cases in Guinea-Bissau between January 1996 and April 1997. Original data from WHO (www.who.int/wer/en/)

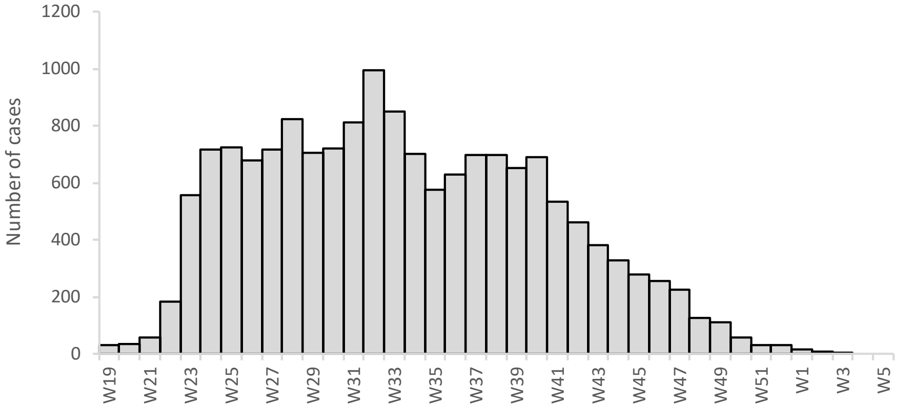

Figure S2. Weekly number of cholera cases in Guinea-Bissau between May 1997 and January 1998. Original data from WHO (www.who.int/wer/en/)

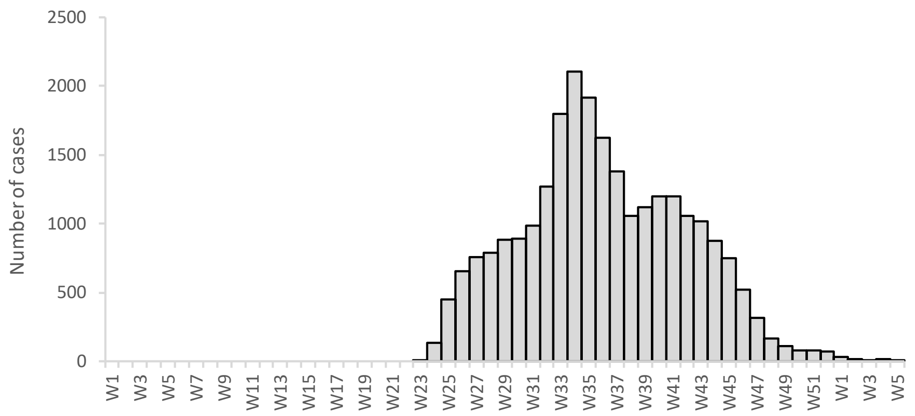

Figure S3. Weekly number of cholera cases in Guinea-Bissau between June 2005 and February 2006. Original data from WHO (www.who.int/wer/en/)

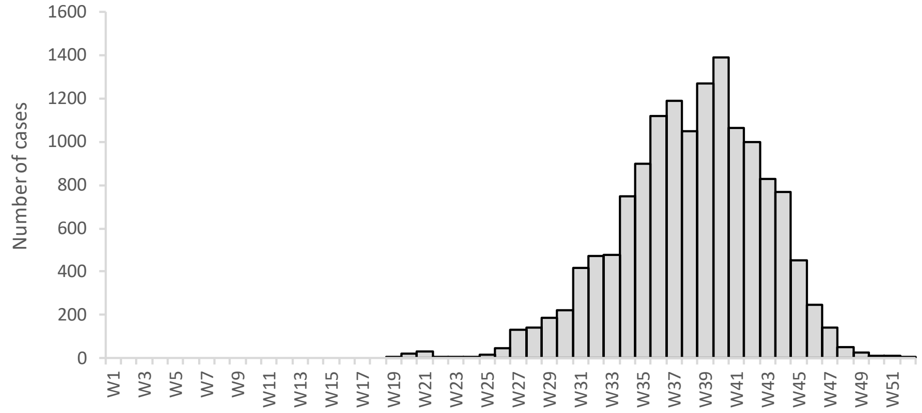

Figure S4. Weekly number of cholera cases in Guinea-Bissau between May 2008 and January 2009. Original data from WHO (www.who.int/wer/en/)

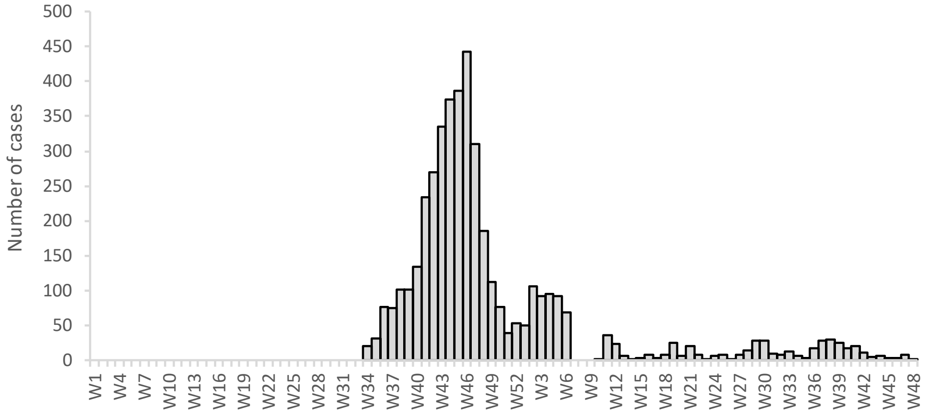

Figure S5. Weekly number of cholera cases in Guinea-Bissau between August 2012 and November 2013. Original data from WHO (www.who.int/wer/en/)

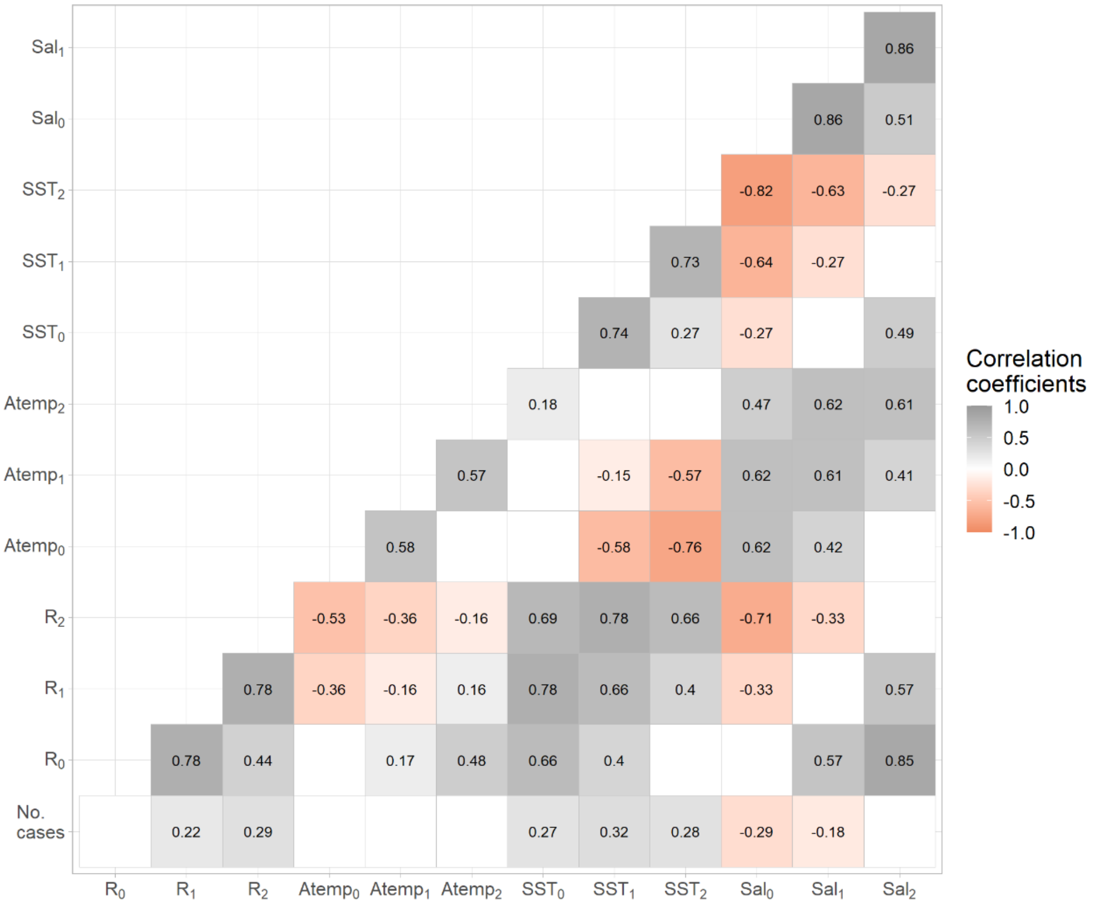

Figure S6. Matrix of Spearman correlations between the monthly number of cholera cases and the environmental variables. All correlations shown are significant at p<0.05. No. cases – number of cholera cases; R – rainfall; Atemp – air temperature; SST – sea surface temperature; Sal – salinity. The environmental variables are referenced with a number indicating their time lag relative to the time when cholera cases were observed (0 – no Lag, 1 – one month prior to the observation of cholera cases observed, 2 – two months prior to the observation of cholera cases).
